# Cardiovascular Health Status, Migraine Risk, and Mortality Outcomes in Migraine Individuals: Insights From NHANES

**DOI:** 10.1002/brb3.71162

**Published:** 2025-12-31

**Authors:** Hongwei Liu, Wenzhi Niu, Gaiying Ma, Haixia Fan, Minheng Zhang, Yu Wang, Mingni Yang

**Affiliations:** ^1^ Department of Neurology Xuanwu Hospital of Capital Medical University Beijing China; ^2^ Department of Neurology, Taiyuan City Central Hospital The Ninth Clinical Medical College of Shanxi Medical University Taiyuan Shanxi Province China; ^3^ Department of Sleep Center First Hospital of Shanxi Medical University Taiyuan Shanxi Province China; ^4^ Department of Gerontology The First People's Hospital of Jinzhong Yuci Shanxi China; ^5^ Department of Emergency Internal Medicine, Shanxi Bethune Hospital, Shanxi Academy of Medical Sciences, Tongji Shanxi Hospital Third Hospital of Shanxi Medical University Taiyuan Shanxi Province China; ^6^ Department of Neurology The Second Affiliated Hospital of Xiamen Medical College Xiamen Fujian Provinece China

**Keywords:** all‐cause mortality, cardiovascular health, cardiovascular mortality, Life's Simple 7, migraine

## Abstract

**Objectives:**

The study analyzes the link between cardiovascular health, measured through the Life's Simple 7 (LS7) score, and the potential risk of migraines. Moreover, the research delves into the possibility that better cardiovascular health might lessen overall and cardiovascular‐related mortality rates in people with migraines.

**Methods:**

Data were drawn from the National Health and Nutrition Examination Survey. Associations between Life's Simple 7 scores and migraine risk were assessed using logistic and restricted cubic spline regressions, with subgroup analyses to examine variations across demographic groups. Cox proportional hazards models and restricted cubic spline regressions evaluated the impact of Life's Simple 7 scores on all‐cause and cardiovascular mortality.

**Results:**

The likelihood of experiencing migraines was significantly lower with higher Life's Simple 7 scores (OR = 0.91, 95% CI: 0.87–0.94, p < 0.001). Those maintaining ideal cardiovascular health were 43% less likely to suffer from migraines than individuals with poor cardiovascular health (OR = 0.57, 95% CI: 0.44–0.74, p < 0.001). Restricted cubic spline analysis suggested a non‐linear inverse connection between Life's Simple 7 scores and migraine risk, while a linear inverse connection was identified between Life's Simple 7 scores and both all‐cause and cardiovascular mortality among migraine individuals. According to Cox proportional hazards models, higher Life's Simple 7 scores correlated with a decrease in all‐cause mortality (HR = 0.88, 95% CI: 0.80–0.97, p = 0.009) and cardiovascular mortality (HR = 0.68, 95% CI: 0.53–0.86, p = 0.002) in individuals with migraines.

**Conclusions:**

The Life's Simple 7 score suggests that maintaining excellent cardiovascular health is connected to a decreased risk of migraines and better survival outcomes for migraine sufferers.

## Graphical abstract

Higher levels of cardiovascular health, as assessed by the Life's Simple 7 score, are associated with a reduced risk of incident migraine and are significantly linked to improved all‐cause and cardiovascular mortality outcomes among individuals with migraine.

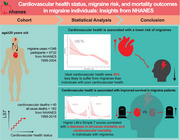

## 1 Introduction

Cardiovascular disease (CVD) is still the primary cause of death worldwide, making it crucial to find effective methods to prevent and lessen its impact on public health. (Khraishah et al. 2022) The framework known as Life's Simple 7 (LS7), introduced by the American Heart Association (AHA), outlines a complete approach to improving cardiovascular health by addressing seven essential factors: smoking, physical activity, diet, body mass index (BMI), blood pressure, cholesterol, and glucose levels. (Lloyd‐Jones et al. 2010) These elements play a key role, both individually and together, in reducing CVD and all‐cause mortality, while also enhancing the overall quality of life. (Arnett et al. 2019, Perak et al. 2020)

Migraine, a debilitating primary headache disorder impacting more than one billion individuals worldwide, is increasingly recognized as a substantial factor in heightened cardiovascular risk. (Kalkman et al. 2023, Ashina et al. 2021) Some research had established a bidirectional association between migraine and cardiovascular outcomes, with a particularly heightened risk evident among individuals with migraine with aura. (Kalkman et al. 2023, Rist et al. 2023) Recent evidence indicates that individuals with migraine, especially those with migraine with aura, are at a heightened risk of myocardial infarction and stroke; moreover, migraine with aura is linked to increased overall cardiovascular mortality. (Ng et al. 2022) The convergence of shared risk factors underlying both migraine and cardiovascular diseases, such as endothelial dysfunction, chronic inflammation, and vascular hyperreactivity, underscores the critical need to examine the relationship between LS7 metrics and migraine outcomes. (Kalkman et al. 2023, Adelborg et al. 2018)

Despite certain advancements, the combined impact of LS7 scores and migraine history on long‐term health outcomes, particularly all‐cause and cardiovascular mortality, remains insufficiently explored. Given the increasing prevalence of migraine and its intricate association with cardiovascular health, evaluating whether optimizing LS7 factors can mitigate the heightened cardiovascular risk among individuals with migraine is essential. The National Health and Nutrition Examination Survey (NHANES), with its wide‐ranging health metrics and detailed follow‐up data, offers a valuable opportunity to investigate these links. (Liu et al. 2023) Previous findings from NHANES have suggested that higher LS7 scores are related to a diminished risk of mortality from all causes and cardiovascular conditions. (Commodore‐Mensah et al. 2022, Chen et al. 2023) Nevertheless, few studies have directly examined whether these associations differ among individuals with a history of migraine. Given that migraineurs, particularly those with aura, frequently present vascular risk factors overlapping with LS7 components, adherence to AHA‐recommended lifestyle modifications may plausibly reshape their risk profile. (Lei et al. 2024) Furthermore, elucidating the interaction between migraine and LS7 metrics could inform more targeted strategies for managing cardiovascular health in this population.

The analytical framework's directionality is a primary distinction between our study and most earlier research. In contrast to past studies that mainly assessed migraine's effect on cardiovascular outcomes, our investigation examines the relationship from the opposite direction. Specifically, we define cardiovascular health using the American Heart Association's Life's Simple 7 (LS7) metric as the exposure and analyze its connection to both migraine risk and long‐term mortality. The purpose of the study is to assess the association between LS7 scores and migraine risk and prognosis by utilizing NHANES data from 1999 to 2004, with mortality data from 1999 to 2018 combined via the National Death Index. Specifically, we investigate how cardiovascular health influences migraine risk and further assess whether improvements in cardiovascular health can enhance the prognosis of individuals with migraine. Our analysis seeks to determine if cardiovascular health metrics, effective in the general population, confer similar benefits for individuals with migraine, thereby supporting the development of tailored preventive strategies.

## 2 Materials and Methods

### 2.1 Study Population

**FIGURE 1:**
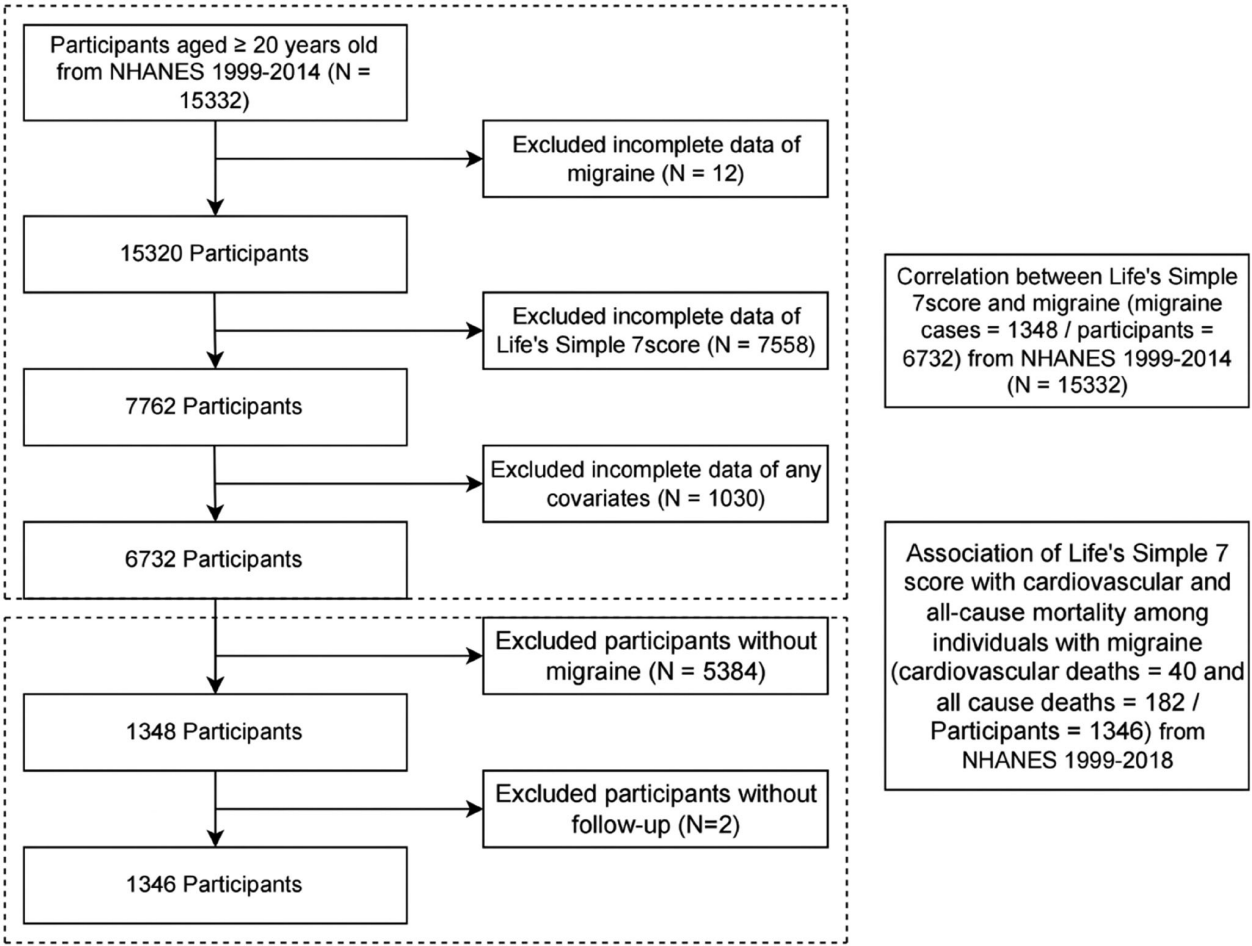
Flow diagram of study cohort selection. **Abbreviation**: NHANES, National Health and Nutrition Examination Survey.

This analysis used NHANES data from the 1999–2000, 2001–2002, and 2003–2004 cycles to investigate the connection between LS7 and the likelihood of migraines. The baseline exposure assessment period for these survey cycles spans from 1999 to 2004. Initially, 15,332 participants aged 20 years or older were enrolled. Following the exclusion of 12 individuals without migraine data, 7558 with incomplete LS7 component data, and 1030 with missing covariates, 6732 participants were included in the cross‐sectional analysis, of which 1348 had migraines (Figure 1). The study on longitudinal mortality did not include participants who were migraine‐free (*n* = 5384). In the group of 1348 migraine cases, 2 individuals were without mortality follow‐up data, leading to 1346 participants with migraines. Through probabilistic linkage to the National Death Index, mortality status was determined, with follow‐up occurring from the interview date until the end of 2018 (Figure 1). This offered a follow‐up timeframe of approximately 20 years.

### 2.2 Assessment of LS7 Score

According to the AHA, the LS7 score includes seven components: smoking status, physical activity, diet, BMI, blood pressure, total cholesterol, and blood glucose levels. (Elgazzar et al. 2020) Based on established thresholds, components were rated as poor (0 points), intermediate (1 point), or ideal (2 points). (Supplementary Table S1). By summing the scores of each component, the overall LS7 score was obtained, with a potential range from 0 to 14 points. Using their total LS7 scores, participants were sorted into categories of poor (0–4), intermediate (5–9), or ideal (10–14) cardiovascular health. (Hasbani et al. 2022) Data regarding dietary intake were sourced from 24‐hour dietary recall interviews. To accommodate potential variations between weekdays and weekends, the recalls were weighted according to the day of the week, as guided by NHANES. Participants with implausible total energy intake (<500 or >5000 kcal/day for women; <800 or >8000 kcal/day for men) were excluded to reduce measurement error. Established scoring algorithms were used to compute HEI‐2015 component scores, and the HEI score was then categorized according to LS7 diet classification thresholds (Supplementary Materials). (Rehm et al. 2016, Reedy et al. 2018)

### 2.3 Assessment of Migraine and Mortality Ascertainment

To identify migraine patients, the inclusion criteria were drawn from the pain section of the NHANES self‐assessment questionnaire. Participants' migraine status was identified through the NHANES self‐report question regarding ‘severe headache or migraine’ in the preceding 3 months. This measure is often used in NHANES migraine research but does not correspond to a clinical or ICHD‐confirmed diagnosis. (Li et al. 2024, Zhang et al. 2024) This item has been a long‐standing instrument for NHANES in tracking the burden of severe headache disorders within the population. Its design reflects a balance between epidemiological efficiency and measurement reliability: as a brief screening tool, it identifies individuals who both sought professional medical evaluation for headache‐related symptoms and continued to experience clinically relevant manifestations in the recent past. Although the measure does not differentiate among specific headache subtypes (e.g., migraine vs. tension‐type headache), it consistently captures a subgroup characterized by distinctive patterns of healthcare utilization, functional impairment, and disease burden.

The NHANES Public‐Use Linked Mortality Files, spanning from 1999 to 2018, were compiled by the National Center for Health Statistics using data from the National Death Index. The primary endpoints analyzed were CVD mortality and all‐cause mortality. Tracking started on the NHANES interview date and lasted until the participant's death or December 31, 2019. The definition of cardiovascular mortality relied on the underlying cause‐of‐death codes from the National Death Index. ICD‐10 codes I00‐I09, I11, I13, I20‐I51, and I60‐I78 were used to identify deaths from cardiovascular disease, following the definitions applied by NCHS and previous NHANES‐based mortality studies (Supplementary Materials).

### 2.4 Covariates

Established literature and clinical knowledge guided the selection of covariates. Covariates included age, gender, race, marital status, educational level, drinking status, poverty‐income ratio (PIR), and CVD status. Supplementary Table S2 contains additional information regarding these covariates.

### 2.5 Statistical Analysis

The analyses included the complex, multistage sampling design of NHANES. Survey strata were specified using SDMVSTRA, primary sampling units with SDMVPSU, and examination weights were applied because LS7 contains lab‐derived components. The MEC examination weight (WTMEC2YR) was used for each two‐year cycle, and for the combined 1999–2004 sample, a six‐year weight was calculated by dividing WTMEC2YR by three, according to the National Center for Health Statistics' advice (Supplementary Materials). These weights were employed in all descriptive and inferential analyses to ensure that the estimates are indicative of the U.S. civilian, non‐institutionalized population and that the standard errors accurately mirror the survey design. Survey‐weighted means and standard errors summarized continuous variables, and survey‐weighted frequencies and percentages represented categorical variables. In light of the missing covariate values, we performed multiple imputation using chained equations to mitigate bias and prevent efficiency loss. Included in the imputation model were all the variables from the analytic models, covering demographic, socioeconomic, lifestyle, clinical, and LS7‐related aspects. In R, the mice package was utilized to carry out multiple imputation by chained equations, under the assumption that the missing data mechanism was missing at random. Incorporating all analytic covariates and relevant auxiliary variables, the imputation model aimed to enhance estimation efficiency and reduce potential bias. Five datasets were created through imputation, with each dataset undergoing 50 iterations to ensure the chained equations converged properly. The imputation of continuous variables was done using predictive mean matching, binary variables were handled with logistic regression, nominal categorical variables with multinomial models, and ordinal variables with proportional‐odds models.

We evaluated the proportional hazards assumption using global and covariate‐specific Schoenfeld residual tests. The statistical tests did not reveal any meaningful deviation from proportionality, indicating that the proportional hazards assumption was reasonably satisfied. For continuous variables, between‐group differences were evaluated using survey‐weighted Kruskal–Wallis tests, and for categorical variables, survey‐weighted chi‐square tests were used. We fitted multivariable survey‐weighted logistic regression models to analyze the association between cardiovascular health and migraine, using the LS7 score as the main exposure, modeled as both a continuous and categorical variable (poor, intermediate, and ideal cardiovascular health). Three models were constructed: Model 1 was unadjusted; Model 2 was adjusted for age, gender, race/ethnicity, and educational level; and Model 3 included further adjustments for marital status, poverty‐income ratio (PIR), drinking status, and CVD status. The LS7 score calculation has already accounted for clinical parameters, including diabetes, hypertension, obesity, and dyslipidemia, making further adjustments unnecessary. We used survey‐weighted restricted cubic spline (RCS) regression to characterize potential non‐linear associations between LS7 score and migraine risk in the cross‐sectional analysis and between LS7 score and all‐cause and cardiovascular mortality among individuals with migraine. Knots were specified at four points: the 5th, 35th, 65th, and 95th percentiles of the LS7 score distribution. Reported were the p‐value for the non‐linear component and the approximate point of inflection. Effect modification was assessed by analyzing clinically significant subpopulations through subgroup and interaction analyses. In the longitudinal analysis restricted to participants with migraine, survival curves for all‐cause and cardiovascular mortality across LS7 categories were estimated using survey‐weighted Kaplan‐Meier methods, and differences between groups were evaluated with survey‐weighted log‐rank tests. Survey‐weighted Cox proportional hazards models were then used to estimate hazard ratios and 95% confidence intervals for mortality outcomes associated with LS7 score, treated as both a continuous and a categorical variable, with the same sequence of covariate adjustment as in the logistic models. For all statistical analyses, R software version 4.4.1 from the R Foundation for Statistical Computing in Vienna, Austria, was employed. Survey‐weighted methods were applied using specific functions designed for complex survey data. A two‐sided p‐value of less than 0.05 was used to establish statistical significance.

## 3 Results

### 3.1 Baseline Characteristics of Study Participants

In Supplementary Table S3, the starting characteristics of 6,732 participants are outlined, sorted by their history of migraines. Individuals with a history of migraines (*n* = 1348) were, on average, younger than those without (40.61 ± 0.40 vs. 46.21 ± 0.38 years, *p* < 0.001) and had a higher percentage of females (62.24% vs. 44.72%, p < 0.001). The study observed significant racial/ethnic differences, with a reduced proportion of non‐Hispanic White participants among migraine sufferers compared to non‐sufferers (75.06% vs.79.44%, p = 0.047). In addition, people with a migraine history had achieved less in terms of education (p = 0.002) and had higher poverty rates (p < 0.001) than those who did not suffer from migraines. In terms of marital status (p = 0.136) and CVD prevalence (p = 0.457), no significant differences were found between the two groups.

For the LS7 components, individuals with migraines scored lower in smoking (p = 0.005), BMI (p = 0.006), and dietary intake (p < 0.001), yet had higher scores in blood pressure (P<0.001), blood glucose (p = 0.007), and cholesterol (p = 0.026). Individuals suffering from migraines did not show significant differences in physical activity (p = 0.868) or total LS7 score (p = 0.231).

### 3.2 Association Between LS7 Score and Migraine

The association between LS7 scores and migraine, as analyzed through multivariable logistic regression, is detailed in Table 1. Model 1, which was not adjusted, showed that LS7 scores were not significantly related to migraine (OR = 0.98, 95% CI: 0.96–1.01, p = 0.228). Upon adjusting for factors such as age, gender, race/ethnicity, and education level (Model 2), elevated LS7 scores were connected to a reduced risk of migraine (OR = 0.91, 95% CI: 0.87–0.94, p < 0.001). After additional adjustments for marital status, PIR, drinking status, and CVD history in Model 3, the association still showed significance (OR = 0.92, 95% CI: 0.88–0.96, p < 0.001).

**TABLE 1:** Weighted multivariate logistic regression analysis of Life's Simple 7 score and migraine^a^

Life's Simple 7 score	Model 1 OR (95% CI), *p*‐value	Model 2 OR (95% CI), *p*‐value	Model 3 OR (95% CI), *p*‐value
Continuous	0.98 (0.96, 1.01), 0.228	0.91 (0.87, 0.94), < 0.001	0.92 (0.88, 0.96), < 0.001
Categorical			
Poor	Reference	Reference	Reference
Intermediate	1.01 (0.86, 1.18), 0.936	0.83 (0.69, 1.00), 0.048	0.86 (0.72, 1.03), 0.100
Ideal	0.83 (0.68, 1.01), 0.061	0.54 (0.43, 0.69), <0.001	0.57 (0.44, 0.74), < 0.001
P for trend	0.106	< 0.001	< 0.001

^a^ Model 1: unadjusted; Model 2: adjusted for age, sex, race, and educational attainment; Model 3: adjusted for age, sex, race, educational attainment, marital status, PIR, drinking status, and CVD .

*Note*: Values are presented as unweighted sample sizes and survey‐weighted percentages. All estimates account for NHANES sampling strata, clusters, and MEC examination weights for the 1999‐2004 combined cycles.

**Abbreviations**: CI, confidence intervals; CVD, cardiovascular disease; OR, odds ratios; PIR, family poverty income ratio.

The fully adjusted model, which analyzed LS7 as a categorical variable, showed that participants with ideal cardiovascular health had a significantly reduced risk of migraines compared to those with poor cardiovascular health (OR = 0.57, 95% CI: 0.44–0.74, p < 0.001) (Model 3). The analysis of trends also showed a significant negative association between LS7 categories and migraine risk (trend p < 0.001).

### 3.3 Association Between LS7 Component Scores and Migraine

The analyses of multivariable logistic regression for each LS7 component are displayed in Table 2. The fully adjusted Model 3 showed that increased scores for smoking (OR = 0.87, 95% CI: 0.78–0.98, p = 0.019), BMI (OR = 0.85, 95% CI: 0.76–0.95, p = 0.005), and dietary intake (OR = 0.76, 95% CI: 0.66–0.87, p < 0.001) were significantly correlated with a reduced likelihood of migraine. However, physical activity, blood pressure, blood glucose, and cholesterol scores were not significantly associated with migraine in the fully adjusted model (all p > 0.05).

**TABLE 2:** Weighted multivariate logistic regression analysis of Life's Simple 7 components score and migraine^a^

Life's Simple 7 components	Model 1 OR (95% CI), *p*‐value	Model 2 OR (95%CI), *p*‐value	Model 3 OR (95% CI), *p*‐value
Physical activity score	1.02 (0.84, 1.22), 0.868	1.08 (0.91, 1.28), 0.391	1.08 (0.90, 1.29), 0.413
Smoking score	0.86 (0.78, 0.95), 0.004	0.86 (0.78, 0.95), 0.005	0.87 (0.78, 0.98), 0.019
Blood pressure score	1.30 (1.18, 1.44), < 0.001	0.96 (0.84, 1.10), 0.549	0.96 (0.84, 1.10), 0.566
Body mass index score	0.88 (0.80, 0.96), 0.006	0.83 (0.75, 0.92), < 0.001	0.85 (0.76, 0.95), 0.005
Glucose score	1.15 (1.04, 1.27), 0.009	0.90 (0.82, 1.00), 0.047	0.94 (0.85, 1.04), 0.230
Cholesterol score	1.10 (1.01, 1.20), 0.028	0.93 (0.85, 1.02), 0.113	0.93 (0.85, 1.03), 0.143
Dietary intake score	0.64 (0.57, 0.72), < 0.001	0.73 (0.64, 0.83), < 0.001	0.76 (0.66, 0.87), < 0.001

^a^ Model 1: unadjusted; Model 2: adjusted for age, sex, race, and educational attainment; Model 3: adjusted for age, sex, race, educational attainment, marital status, PIR, drinking status, and CVD.

*Note*: Values are presented as unweighted sample sizes and survey‐weighted percentages. All estimates account for NHANES sampling strata, clusters, and MEC examination weights for the 1999‐2004 combined cycles.

**Abbreviations**: CI, confidence intervals; CVD, cardiovascular disease; OR, odds ratios; PIR, family poverty income ratio.

### 3.4 Nonlinear Association Between LS7 Score and Migraine

**FIGURE 2:**
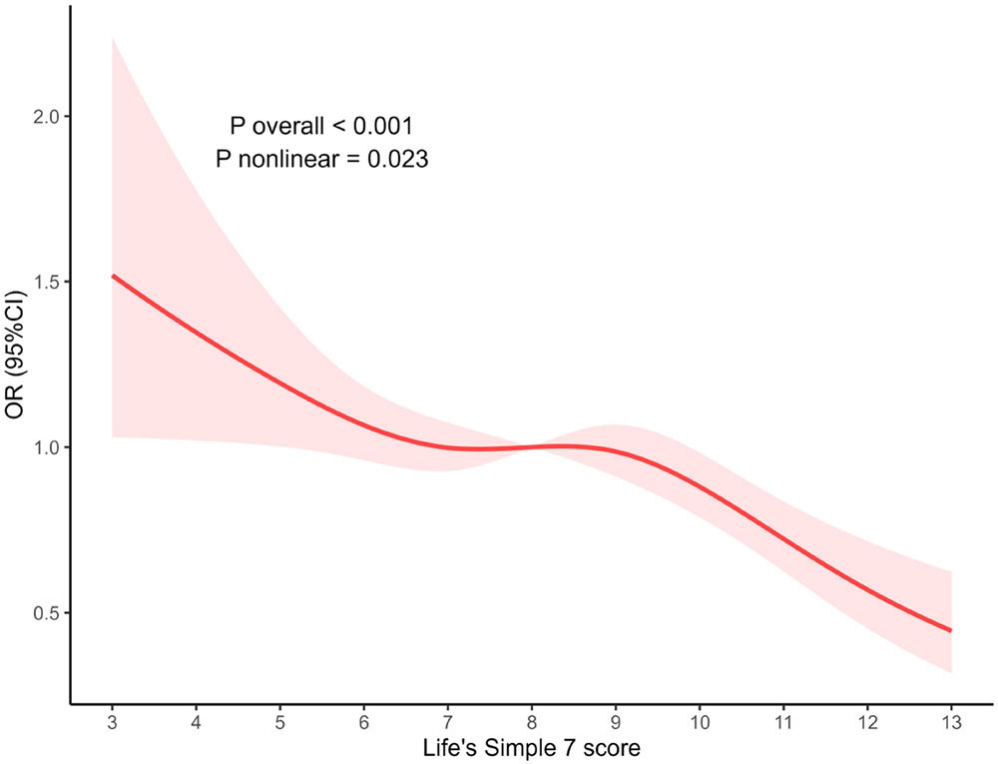
Dose‐response relationships between Life's Simple 7 score and migraine Adjustment factors included age, sex, race, educational attainment, marital status, PIR, drinking status, and CVD. **Abbreviations**: CI, confidence intervals; CVD, cardiovascular disease; OR, odds ratios; PIR, family poverty income ratio.

The analysis using RCS indicated a nonlinear relationship between the risk of migraines and LS7 scores (Figure 2). After adjustments for age, gender, race/ethnicity, educational level, marital status, PIR, drinking status, and CVD history, this association remained significant (non‐linear p < 0.001). The findings suggested that as LS7 scores rose, the negative link with migraine risk became more pronounced, hinting that maintaining optimal cardiovascular health could offer additional defense against migraines. By visually inspecting the spline curve, it is evident that the main inflection point occurs at an LS7 score near 7, where the OR approaches 1.0. A secondary fluctuation of smaller magnitude is detected around LS7 scores of 9 to 10, succeeded by a further decline toward the lowest‐risk range for scores above 12. Between LS7 scores of 3 and 6, OR values drop most steeply, falling from approximately 1.5 to about 1.05. The smallest estimated OR, roughly 0.45 to 0.50, occurs at LS7 scores of 13.

### 3.5 Stratified and Interaction Analysis

Subgroup analyses explored whether the correlation between LS7 scores and migraines was different across age, gender, race, education level, marital status, PIR, drinking status, and CVD status (Supplementary Figure S1). In all subgroups, interaction *p*‐values were over 0.05, which implies no significant differences in the LS7‐migraine connection across these demographic and health‐related groups.

### 3.6 Kaplan‐Meier Analysis

The study of 1346 migraine sufferers, observed over a median follow‐up of 208 months, reported 182 deaths from all causes and 40 deaths specifically from cardiovascular conditions. Kaplan‐Meier survival curves, as presented in Supplementary Figure S2, highlight the protective impact of elevated LS7 scores on reducing both all‐cause and cardiovascular mortality among those suffering from migraines. Using log‐rank tests, significant differences in survival rates were observed across LS7 score categories for both all‐cause and cardiovascular mortality (p < 0.001).

### 3.7 Association Between LS7 Score and Mortality in Migraine Patients

**FIGURE 3:**
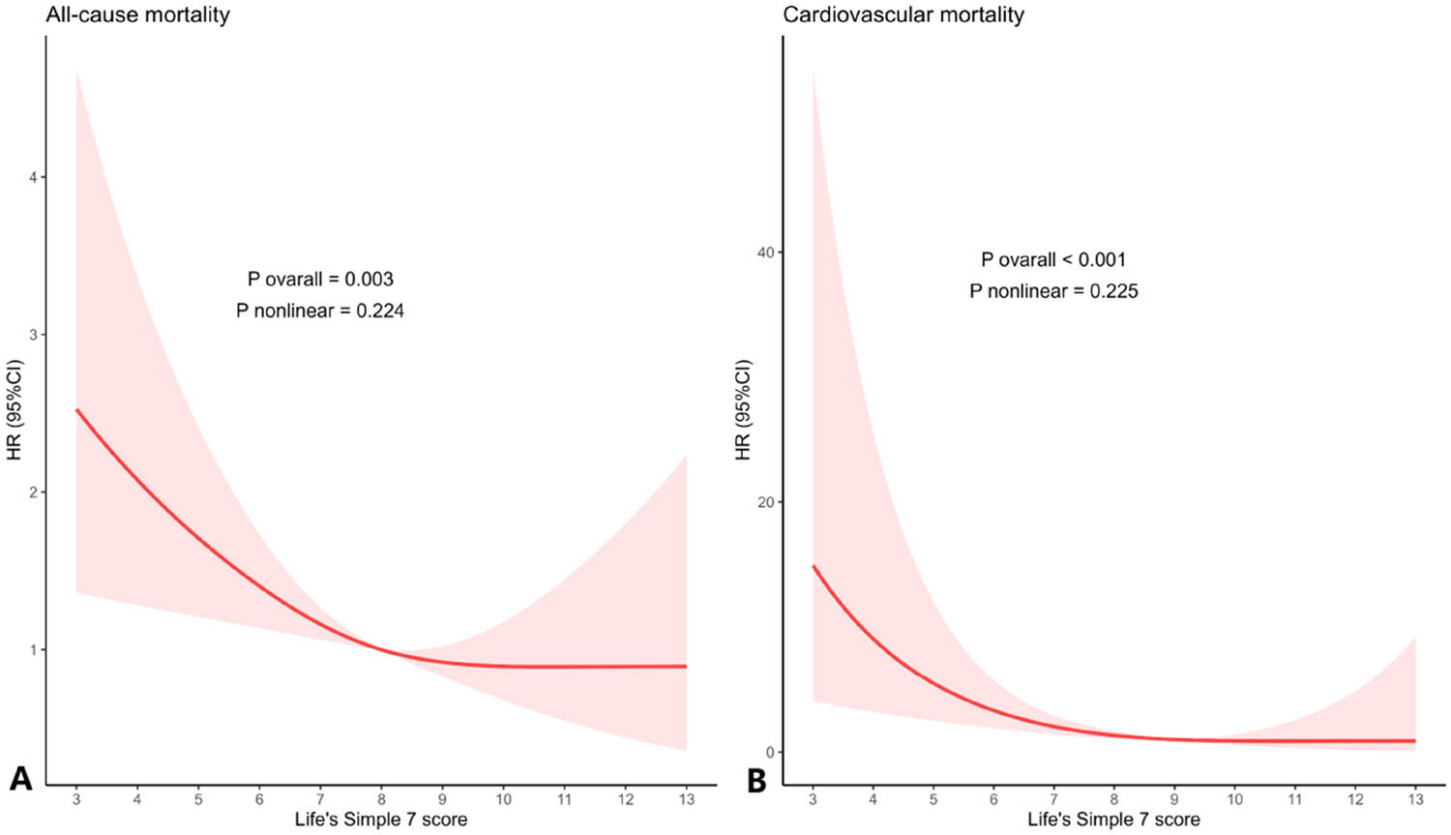
Association between Life's Simple 7 score and all‐cause (**A**) and cardiovascular mortality (**B**) in migraine patients Adjusted for age, sex, race, and educational attainment, marital status, PIR, drinking status, and CVD. **Abbreviations**: CI, confidence intervals; CVD, cardiovascular disease; OR, odds ratios; PIR, family poverty income ratio.

In Table 3, the findings from Cox proportional hazards models are displayed, examining the relationship between LS7 scores and mortality among those with migraines. In the poor LS7 group, 23 cardiovascular deaths were recorded, compared to 12 in the intermediate group and 5 in the ideal group. The fully adjusted model indicated that higher LS7 scores were significantly linked to a reduced risk of mortality from all causes (HR = 0.88, 95% CI: 0.80–0.97, p = 0.009) and cardiovascular mortality (HR = 0.68, 95% CI: 0.53–0.86, p = 0.002). The risk of cardiovascular mortality was significantly lower in individuals with ideal cardiovascular health compared to those with poor cardiovascular health (HR = 0.05, 95% CI: 0.01–0.31, p < 0.001). Figure 3’s RCS analysis further demonstrated a linear inverse connection between LS7 scores and mortality, with no nonlinear evidence (all non‐linear p > 0.05). Across the spectrum of LS7 scores, the restricted cubic spline curve demonstrated a clear inverse association with all‐cause mortality. With an increase in LS7, the hazard ratio decreased significantly, especially between scores 3 and 7, where it went from about 2.4 to nearly 1.0. The curve stabilized between LS7 scores of 7 and 9, reflecting a period where the mortality risk was relatively unchanged. Even at LS7 levels of 10 or higher, the HR was maintained below 1.0, but the confidence intervals widened as there were fewer participants with optimal cardiovascular health. The risk of cardiovascular mortality dropped significantly as LS7 scores rose from 3 to 7, with the hazard ratio falling from about 2.8 to roughly 1.0. From scores 7 to 9, the decline in the curve leveled off. With LS7 levels at 10 or higher, the HR was near or slightly under 1.0, but the confidence intervals widened as only a few participants attained the highest cardiovascular health scores.

**TABLE 3:** Multivariate Cox regression analysis of Life's Simple 7 score and all‐cause and cardiovascular mortality among individuals with migraine^a^

Life's Simple 7 score	Model 1 HR (95% CI), p‐value	Model 2 HR (95% CI), p‐value	Model 3 HR (95% CI), p‐value
**All‐cause mortality**			
Continuous	0.78 (0.72, 0.85), < 0.001	0.85 (0.77, 0.94), 0.002	0.88 (0.80, 0.97), 0.009
Categorical			
Poor	Reference	Reference	Reference
Intermediate	0.48 (0.34, 0.68), < 0.001	0.61 (0.43, 0.87), 0.007	0.67 (0.47, 0.96), 0.029
Ideal	0.31 (0.17, 0.55), < 0.001	0.51 (0.26, 0.98), 0.044	0.62 (0.33, 1.17), 0.138
P for trend	< 0.001	0.005	0.024
**Cardiovascular mortality**			
Continuous	0.64 (0.53, 0.77), < 0.001	0.68 (0.53, 0.88), 0.003	0.68 (0.53, 0.86), 0.002
Categorical			
Poor	Reference	Reference	Reference
Intermediate	0.35 (0.17, 0.74), 0.006	0.43 (0.20, 0.93), 0.032	0.42 (0.20, 0.89), 0.023
Ideal	0.03 (0.00, 0.23), < 0.001	0.05 (0.01, 0.34), 0.002	0.05 (0.01, 0.31), < 0.001
P for trend	< 0.001	0.002	0.001

^a^ Model 1: unadjusted; Model 2: adjusted for age, sex, race, and educational attainment; Model 3: adjusted for age, sex, race, educational attainment, marital status, PIR, drinking status, and CVD.

*Note*: Values are presented as unweighted sample sizes and survey‐weighted percentages. All estimates account for NHANES sampling strata, clusters, and MEC examination weights for the 1999–2004 combined cycles.

**Abbreviations**: CI, confidence intervals; CVD, cardiovascular disease; HR Hazard ratio; PIR, family poverty income ratio.

## 4 Discussion

In this study of U.S. adults that is representative on a national scale, higher LS7 scores, indicating better cardiovascular health, were significantly linked to a lower likelihood of migraines and reduced risks of all‐cause and cardiovascular mortality. The correlation between LS7 scores and migraine risk was nonlinear, suggesting that improvements in cardiovascular health could lead to increasingly greater migraine protection. Even after adjusting for a range of demographic, behavioral, and metabolic covariates, these associations remained solid. Together, the findings imply that maintaining optimal cardiovascular health behaviors and factors may aid in preventing migraines and improving long‐term survival, highlighting the strong link between vascular and neurological health. Differences between component‐specific estimates and the composite LS7 score may arise from several factors. First, the relationships among LS7 components can cause collinearity, affecting the precision of component‐level estimates. Furthermore, the components are measured on different scales and may not contribute equally to the overall distribution. Third, LS7 functions as a combined metric that more effectively represents joint cardiovascular health than any single component, potentially leading to differences between the composite and individual associations. For these reasons, component‐level findings are interpreted as exploratory.

Our findings support the expanding literature on the connection between migraines and cardiovascular health, while extending the evidence in unique ways. Large‐scale meta‐analytic and cohort research reveals that people with migraines, especially women and those with aura, have a heightened risk of ischemic stroke and other vascular outcomes. (Liu et al. 2023, Schürks et al. 2009, Huang et al. 2025) Further data from a national registry suggest broader rises in heart attacks, hemorrhagic strokes, venous blood clots, and atrial fibrillation. (Adelborg et al. 2018) Findings on mortality have been varied, with some groups indicating elevated cardiovascular and all‐cause mortality in cases of migraine with aura (Gudmundsson et al. 2010), whereas others suggest attenuation after multivariable adjustment; within this context, our observation that higher LS7 scores are associated with lower all‐cause and cardiovascular mortality among individuals with migraine extends prior work by focusing on modifiable cardiovascular health rather than migraine status alone. (Liu et al. 2023) Moreover, previous investigations have examined individual LS7 components such as hypertension, diabetes, diet, body mass index, smoking, and adiposity; the reported associations with migraine have been inconsistent, with some factors showing positive, negative, or even bidirectional relationships. (Xiao et al. 2025, Zhuang et al. 2024, Wu et al. 2023, Antonazzo et al. 2018, Tian et al. 2024, Amin et al. 2018, Nguyen and Schytz 2024) In this case, utilizing the composite LS7 framework offers a more inclusive perspective that addresses both the behavioral and biological aspects of cardiovascular health. The nonlinear association between LS7 and migraine risk that we observed complements population data linking better cardiovascular health scores to fewer vascular events and supports the hypothesis that gains at higher levels of overall cardiovascular health may confer disproportionate benefit for migraine as well. By comparing these factors, it becomes evident that aligning migraine prevention with cardiovascular health principles enhances its value beyond just noting comorbid risks and highlights LS7‐based targets as useful mechanisms for improving both neurological and cardiovascular health.

Multiple biological mechanisms may underlie the observed link between cardiovascular health and migraine. The onset and propagation of migraines are influenced by impaired cardiovascular health, which causes endothelial dysfunction, reduced nitric oxide bioavailability, and abnormal cerebrovascular reactivity. (Ashina et al. 2021, Marichal‐Cancino et al. 2021) Cortical spreading depolarization is promoted by endothelial dysfunction and microvascular instability, which also compromise the blood‐brain barrier and increase the sensitivity of trigeminovascular afferents. (Charles 2018, Hoffmann et al. 2019, Silvestro et al. 2024, Nixdorf et al. 2008) Individuals with poorer LS7 profiles often exhibit elevated systemic inflammation and oxidative stress, which may intensify neurogenic inflammatory responses through increased expression of cytokines such as IL‐6, TNF‐α, and CRP, and contribute to greater vascular stiffness. (Ulutaş et al. 2025, Chen et al. 2022, Yamanaka et al. 2021, Li et al. 2025, Vuralli et al. 2024) Conditions such as obesity, insulin resistance, and dyslipidemia, which are metabolic disturbances, can further trigger pro‐inflammatory and pro‐thrombotic cascades, leading to a higher likelihood of migraine attacks. (Jahromi et al. 2023, Bhoi et al. 2012, Cavestro 2025) Moreover, current research indicates a dysregulation in the gut‐brain axis, where diet quality and microbiota composition play a role in endothelial health, neuroinflammation, and pain management. (Zhang et al. 2025, Zhou et al. 2024, Gorenshtein et al. 2025) On the other hand, implementing healthy practices that increase LS7 scores, including regular workouts, an improved diet, and stopping smoking, can boost vascular compliance, influence autonomic tone, and diminish neuroinflammatory impact. (Hanssen et al. 2017, Hahad et al. 2021, Higashi 2023, Behrouz et al. 2025, Feyzpour et al. 2024) Taken together, these mechanistic links indicate that enhancing cardiovascular health could lessen the burden of migraine by influencing both vascular function and neurobiological pathways. Within this context, optimizing LS7 emerges as a practical target for prevention and intervention, supporting a more integrated approach to neuro‐cardiovascular health management.

From the viewpoint of public health and clinical practice, the results point to the need for merging cardiovascular health promotion with migraine prevention and management strategies. Healthy diet, sustained physical activity, appropriate weight maintenance, avoidance of smoking, and effective control of blood pressure, lipids, and glucose represent the essential components of the LS7 construct. These factors function not only as foundational strategies for preventing cardiovascular disease but also as modifiable influences that can affect the severity and progression of migraine. (Quartetti et al. 2025, Seng et al. 2022) Structured lifestyle programs delivered in primary care and neurology settings, which encompass sleep optimization, dietary improvement, increased physical activity, and stress reduction, have been shown to meaningfully reduce migraine frequency and disability. These behavioral strategies also represent key components of modern approaches to cardiovascular risk reduction. (Sacco et al. 2012, Agbetou and Adoukonou 2022, Deng et al. 2024) Public health initiatives that promote population‐level adherence to LS7 goals, including community‐based behavioral programs and policy measures targeting smoking and nutritional environments, may therefore generate benefits that extend across both neurological and cardiovascular health domains. (Lei et al. 2024, Hill et al. 2023)

Despite offering valuable insights into the relationship between cardiovascular health and migraines, the study has limitations. The NHANES cross‐sectional design limits our ability to infer causality between LS7 scores and migraines. First, the use of a self‐reported measure to identify migraine, rather than an ICHD‐based clinical diagnosis, may lead to non‐differential misclassification and potentially weaken the true associations. Second, although the AHA has transitioned from LS7 to the more comprehensive LE8 framework, LS7 remained the appropriate metric for our study because essential LE8 components, such as sleep health, non‐HDL cholesterol, and detailed nicotine exposure measures, were not consistently captured in NHANES 1999–2004. Consequently, valid operationalization of LE8 was not feasible for this dataset. Furthermore, the use of LS7 boosts comparability with earlier epidemiologic findings and migraine studies that primarily depended on LS7. Recent research has demonstrated that LS7, although simpler in its scoring system, matches LE8 in predictive performance for mortality outcomes. (Zhu et al. 2025) Future investigations should seek to confirm these findings in longitudinal studies and delve into the mechanisms connecting LS7 components with migraine. Future studies should strive to confirm these results in longitudinal research and investigate how LS7 components are connected to migraines. Third, the ideal group, along with other LS7 categories, experienced a limited number of cardiovascular deaths. Thus, the hazard ratio comparing ideal to poor LS7 is based on a limited number of events and should be interpreted with caution, as it may be prone to instability due to insufficient data. Fourth, the analyses of the LS7 components individually were underpowered, possibly resulting in estimates that lack precision. Hence, these associations at the component level are exploratory, and no conclusive statements about their individual effects are provided. Overall, these limitations highlight the necessity for future research with a larger number of cardiovascular events to more comprehensively evaluate the prognostic role of LS7 in individuals experiencing migraines. Fifth, despite significant covariate adjustments, some degree of unmeasured confounding is unavoidable in observational research, and factors not captured in NHANES might introduce a small bias. If existing conditions alter lifestyle behaviors that contribute to LS7 scores, reverse causality is also a possibility. However, the consistency, strength, and graded nature of the associations observed in this study, together with their alignment with findings from prior cohort research, suggest that these limitations should be interpreted with caution but are unlikely to alter the overall direction of the main associations.

## 5 Conclusions

In essence, the AHA's LS7 for maintaining cardiovascular health was associated with a lower incidence of migraines and better survival prospects. These findings highlight the shared pathways between cardiovascular and neurological health and suggest that promoting LS7‐based lifestyle strategies may help prevent migraine and enhance long‐term outcomes. Further investigations should aim to uncover the basic mechanisms and examine combined prevention methods that focus on managing both cardiovascular issues and migraines.

## Author Contributions

The study's conception and design were contributed to by L. H, W., M. G. Y., F. H. X., and Z. Y. L. H. W., M. G. Y., Z. M. H., and N. W. Z. are accountable for extracting data, analyzing it, visualizing the results, and writing the manuscript. L. H. W., F. H. X., N. W. Z., and Z. Y. handled the manuscript's review and revision.

## Funding

The study was backed by the Basic Research Program of Shanxi Province (Grant No: 20210302124579) and received further funding from the Taiyuan Bureau of Science and Technology, Science, Technology, and Innovation Medical Center (Grant No: 202207).

## Ethics Statement

The study was conducted in compliance with the Declaration of Helsinki, following approval by the NHANES Institutional Review Board.

Consent

Written informed consent was obtained from all participations prior to their participation in NHANES.

## Conflicts of Interest

The authors declare no conflicts of interest.

## Supporting information




**Supplementary Materials**: brb371162‐sup‐0001‐SuppMat.docx

**Supplementary Materials**: brb371162‐sup‐0002‐Figures.docx

**Supplementary Materials**: brb371162‐sup‐0003‐Tables.docx

## Data Availability

The data that support the findings of this study are available from the corresponding author upon reasonable request.
