# Supplementary material for: Cardiovascular Health Status, Migraine Risk, and Mortality Outcomes in Migraine Individuals: Insights From NHANES
Source: Brain Behav. 2025 Dec 31;16(1):e71162. doi: 10.1002/brb3.71162 (PMC12755968; doi:10.1002/brb3.71162)
Supplement: Supplementary file 1 — Supplementary Materials: brb371162‐sup‐0001‐SuppMat.docx [file BRB3-16-e71162-s001.docx]

**Supplementary Materials**

**1. Variable definitions**

All variables used in the analyses were derived from NHANES public-use datasets. Age was defined in completed years at the time of examination. Sex was coded as male or female according to NHANES demographic data. Race and ethnicity were categorized following NHANES classifications, including non-Hispanic White, non-Hispanic Black, Mexican American, and other racial or ethnic groups. Education level was defined as less than high school, high school graduate, or more than high school. Marital status was defined as married or living with a partner, previously married, or never married. The poverty-income ratio represented household income relative to the federal poverty threshold.

Smoking status was defined as current smoker or non-smoker based on self-report. Alcohol intake was categorized according to NHANES questionnaire data. Body mass index was calculated as weight in kilograms divided by the square of height in meters. Blood pressure values were based on the average of up to three standardized measurements. Glucose status was determined using fasting plasma glucose and HbA1c values, and lipid parameters included total cholesterol, HDL cholesterol, and triglycerides.

LS7 components were defined using American Heart Association criteria. Diet quality was assessed using HEI-2015 scoring derived from 24-hour dietary recalls. Physical activity was determined based on NHANES physical activity questionnaires and converted to minutes of moderate-equivalent activity. Smoking, BMI, blood pressure, glucose, and lipids were categorized as poor, intermediate, or ideal according to LS7 thresholds. The composite LS7 score was calculated as the sum of the seven components.

Cardiovascular mortality was defined using NHANES-linked mortality data and included ICD-10 codes I00-I09, I11, I13, I20–I51, and I60-I78.

**2. Code Lists and Operational Rules**

Each categorical variable used in the study was generated according to NHANES coding conventions. Race or ethnicity was derived from RIDRETH1. Educational attainment was taken from DMDEDUC2. Smoking status was coded from SMQ020 and SMD030. Alcohol use was based on ALQ101 and ALQ120. Physical activity coding used variables PAD615, PAD630, and related metabolic equivalents. Diet recalls were based on DR1TOT and DR2TOT files. Implausible energy intake values were excluded using thresholds of <500 kcal/day or >5000 kcal/day for women and <800 kcal/day or >8000 kcal/day for men.

For LS7 component scoring, diet was scored using HEI-2015 component algorithms; physical activity scores were determined from weekly moderate-equivalent minutes; BMI used NHANES examination data; blood pressure values used the averaged systolic and diastolic readings; glucose status used LBXGLU and LBXGH; lipids used LBDHDD and LBXTC.

**3. Weight-Combination Rules Across NHANES Cycles**

NHANES employs 2-year examination weights. Because the present study pooled data from three cycles (1999-2000, 2001-2002, and 2003-2004), the MEC examination weight WTMEC2YR was converted to a 6-year weight by dividing WTMEC2YR by three. This approach follows National Center for Health Statistics guidance for generating combined sampling weights in multi-cycle analyses.

**4. Survey design specification and analytic scripts**

The NHANES complex sampling design was specified using SDMVSTRA for stratification and SDMVPSU for clustering. All analyses were performed using survey-weighted procedures to account for the multistage sampling design.

**5. Dietary Processing Procedures**

Dietary intake was assessed using 24-hour dietary recalls from DR1TOT and DR2TOT, applying day-of-week adjustments to account for differences between weekday and weekend dietary patterns. Implausible energy intake values were removed to reduce measurement error. HEI-2015 component scores were generated using standard scoring algorithms, and the LS7 diet component classification used HEI thresholds consistent with American Heart Association LS7 adaptations.
