# Supplementary material for: Cardiovascular Health Status, Migraine Risk, and Mortality Outcomes in Migraine Individuals: Insights From NHANES
Source: Brain Behav. 2025 Dec 31;16(1):e71162. doi: 10.1002/brb3.71162 (PMC12755968; doi:10.1002/brb3.71162)
Supplement: Supplementary file 2 — Supplementary Materials: brb371162‐sup‐0002‐Figures.docx [file BRB3-16-e71162-s002.docx]

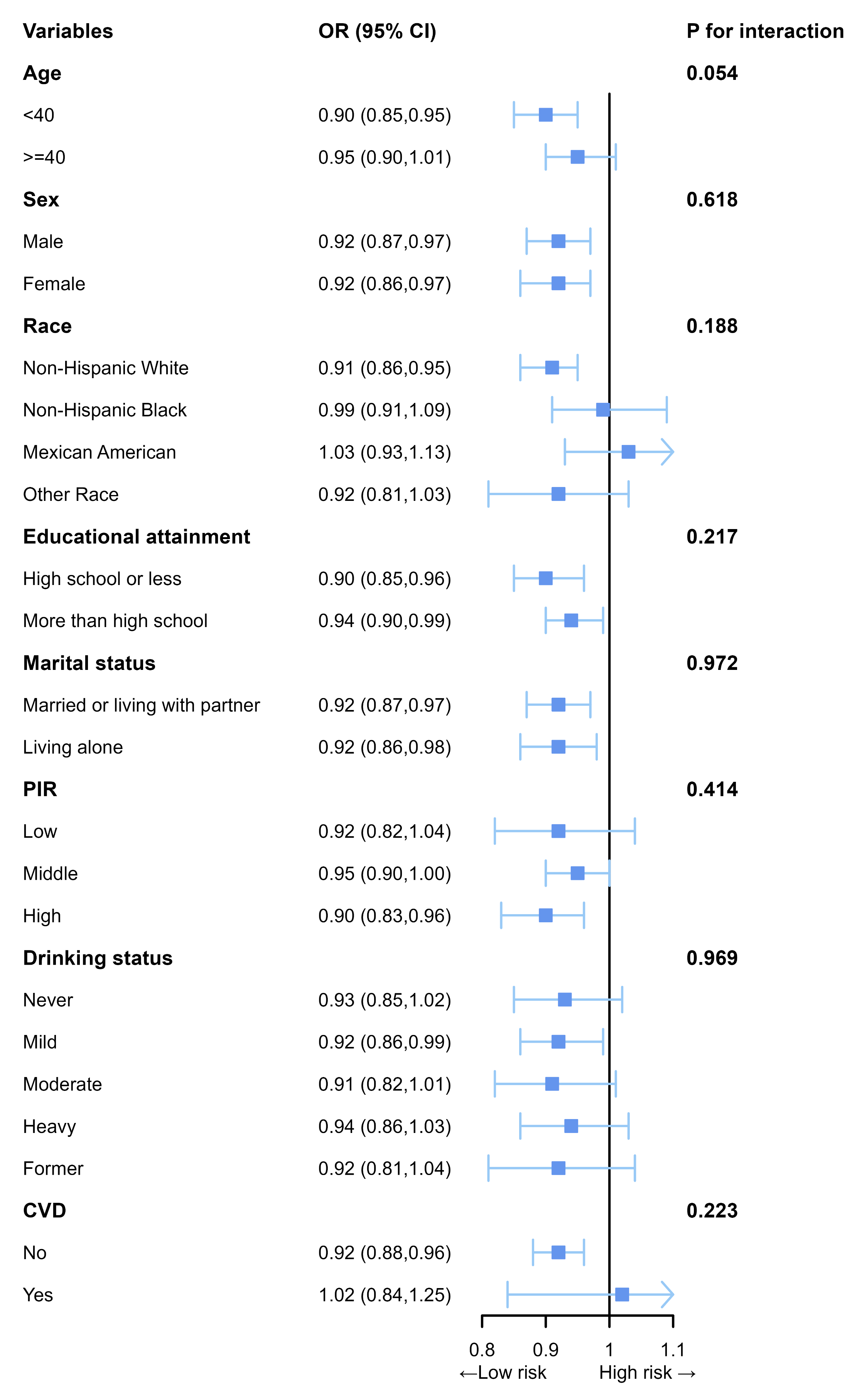


**Supplementary Figure 1** Subgroup analyses by possible effect modifiers for relationship between migraine and Life's Simple 7 score

Analyses were adjusted for covariates age, sex, race, educational attainment, marital status, PIR, drinking status, and CVD when they were not the strata variables

**Abbreviations:** PIR, family poverty income ratio; CVD, cardiovascular disease; OR, odds ratios; CI, confidence intervals


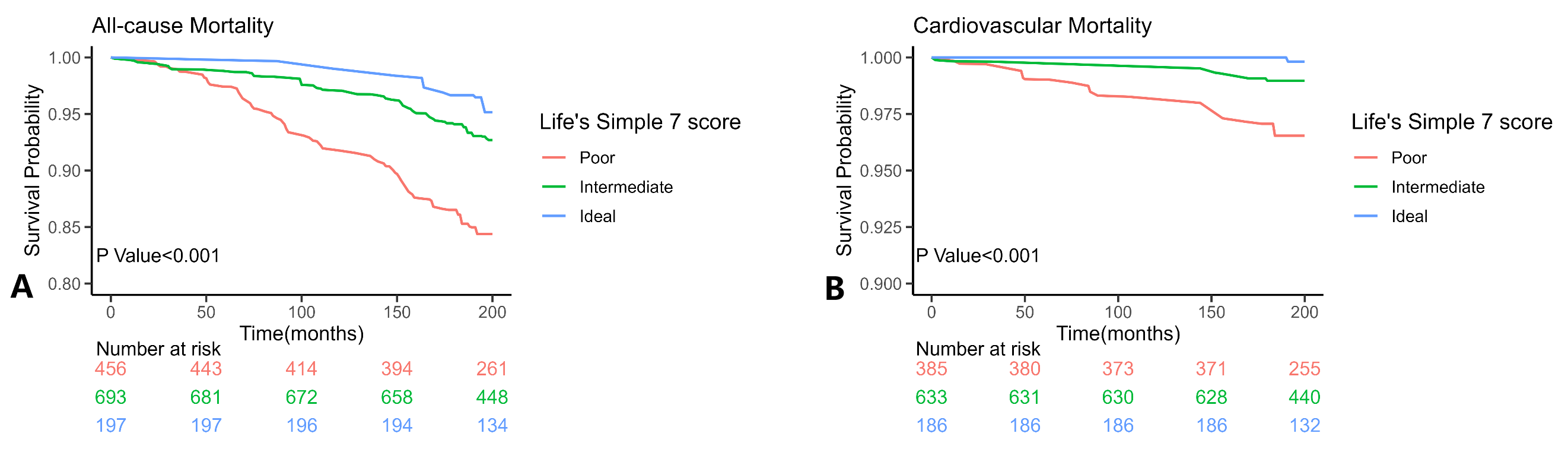


**Supplementary 2** Kaplan-Meier curves were utilized to illustrate the relationship between Life's Simple 7 scores and both all-cause (**A**) and cardiovascular mortality (**B**) among migraine patients
