# Supplementary material for: Cardiovascular Health Status, Migraine Risk, and Mortality Outcomes in Migraine Individuals: Insights From NHANES
Source: Brain Behav. 2025 Dec 31;16(1):e71162. doi: 10.1002/brb3.71162 (PMC12755968; doi:10.1002/brb3.71162)
Supplement: Supplementary file 3 — Supplementary Materials: brb371162‐sup‐0003‐Tables.docx [file BRB3-16-e71162-s003.docx]

**Supplementary Table 1** The Life’s Simple 7 scheme

|  | **Score** | | |
| --- | --- | --- | --- |
|  | **Poor (0)** | **Intermediate (1)** | **Ideal (2)** |
| **Blood pressure** | Treated BP ≥140/90 mm Hg, and BP ≥140/90 mm Hg | SBP 120 to 139 mm Hg or DBP 80 to 89 mm Hg or treated to <120/80 mm Hg | <120/80 mm Hg, without BP-lowering meds |
| **Total cholesterol** | ≥240 mg/dL | 200 to 239 mg/dL or treated to <200 mg/dL | <200 mg/dL, without lipid-lowering medication |
| **Glucose/diabetes** | HbA1c >6.4% | HbA1c 5.7 to 6.4% or treated with insulin or oral meds to HbA1c <5.7% | HbA1c <5.7%, without meds |
| **Smoking** | Current smoker | Former smoker | Never smoker |
| **Body weight** | BMI ≥30 kg/m2 | 25 to 29.9 kg/m2 | <25 kg/m2 |
| **Physical activity** | No activity | 1 to 149 min moderate/vigorous per week | ≥150 min moderate/vigorous per week |
| **Diet** | HEI <50 | HEI 50 to 80 | HEI >80 |

**Note:** In this study, the diet component of Life’s Simple 7 was operationalized using the Healthy Eating Index-2015 (HEI-2015), which aligns with the most recent U.S. Dietary Guidelines and is recommended for NHANES dietary data. HEI-2015 scores were calculated using two 24-hour dietary recalls collected in NHANES. Following standard HEI methodology, dietary intake was quantified using an energy density approach (per 1,000 kcal) rather than absolute consumption. This method reduces confounding by total caloric intake and is consistent with the approach recommended by the U.S. Department of Agriculture.

We used HEI-2015 instead of the original AHA Healthy Diet Score embedded in LS7 for three methodological reasons: 1) NHANES does not provide all components required to compute the traditional AHA Healthy Diet Score, whereas HEI-2015 can be fully constructed from NHANES dietary datasets; 2) HEI-2015 is the most widely validated dietary quality index for NHANES, with extensive documentation, public scoring algorithms, and established reproducibility; 3) HEI-2015 captures a broader range of dietary components, offering a more comprehensive assessment of diet quality in U.S. populations while remaining conceptually consistent with LS7’s emphasis on healthy eating.

**References**

1. Rehm CD, Peñalvo JL, Afshin A, Mozaffarian D. Dietary Intake Among US Adults, 1999-2012. JAMA. 2016 Jun 21;315(23):2542-53. doi: 10.1001/jama.2016.7491.
2. Reedy J, Lerman JL, Krebs-Smith SM, Kirkpatrick SI, Pannucci TE, Wilson MM, Subar AF, Kahle LL, Tooze JA. Evaluation of the Healthy Eating Index-2015. J Acad Nutr Diet. 2018 Sep;118(9):1622-1633. doi: 10.1016/j.jand.2018.05.019

**Abbreviation:** BMI, body mass index; BP, blood pressure; SBP, systolic blood pressure; DBP, diastolic blood pressure; FPG, fasting plasma glucose; HbA1c, hemoglobin A1c; HEI, healthy eating index.

**Supplementary Table 2** The detail definition and classification of covariates

| **Variables** | **Definitions or Classification** |
| --- | --- |
| **Sex** | Male, Female. |
| **Race** | Non-Hispanic White, Non-Hispanic Black, Mexican American, Other Race. |
| **Education attainment** | High school or less, More than high school. |
| **Marital status** | Married or living with partner, Living alone. |
| **PIR** | Low (PIR ≤1), Middle (1< PIR < 4), and High (PIR ≥4). |
| **Smoking status** | Never smoking: <100 cigarettes in lifetime;  Former smoking: >100 cigarettes in life and smoke not at all now;  Now smoking: >100 cigarettes in lifetime. |
| **Drinking status** | Never: consumed fewer than 12 drinks in their lifetime;  Former: consumed at least 12 drinks in one year but did not drink in the last year or did not drink in the last year but had at least 12 drinks in their lifetime;  Heavy: consumed at least 3 drinks per day for females, at least 4 drinks per day for males, or engaged in binge drinking on 5 or more days per month;  Moderate: consumed at least 2 drinks per day for females, at least 3 drinks per day for males, or engaged in binge drinking on at least 2 days per month;  Mild : consumed at most 1 drink per day for females, at most 2 drinks per day for males. |
| **Hypertension** | An average systolic blood pressure (SBP) equal to or exceeding 140 mmHg;  An average diastolic blood pressure (DBP) equal to or exceeding 90 mmHg;  Self-reported hypertension;  Individuals taking prescribed anti-hypertensive medications. |
| **CVD** | The medical conditions section, identified by the variable name prefix MCQ, encompasses self- and proxy-reported personal interview data covering an extensive range of health conditions and medical history for both children and adults. This section includes inquiries such as ‘Has a doctor or other health professional ever informed you/SP that you/he/she… had congestive heart failure, coronary heart disease, angina (also called angina pectoris), heart attack (also called myocardial infarction), stroke, etc.?’ These questions, labeled as MCQ160B-F in the household questionnaires administered during home interviews, were utilized to identify participants with a history of CVD if they responded ‘yes’ to any of these questions. |
| **BMI** | weight (kg)/height (m2). |
| **Laboratory tests** | The Specific method can be found in this webpage  ([https://wwwn.cdc.gov/nchs/nhanes/continuousnhanes/labmethods.aspx?BeginYear=200](https://wwwn.cdc.gov/nchs/nhanes/continuousnhanes/labmethods.aspx?BeginYear=2017)3) |

**Abbreviations:** T2DM, type 2 diabetes mellitus; PIR, family poverty income ratio; CVD, cardiovascular disease; TC, total cholesterol; TG, triglyceride; HDL-C, high-density lipoprotein cholesterol; LDL-C, low-density lipoprotein cholesterol; VAI, visceral adiposity index; BMI, body mass index.

**Supplementary Table 3** Weighted baseline characteristics of participants with or without migraine***^a^***

| **Variables** | **Total** | **History of migraine** | | **P value** |
| --- | --- | --- | --- | --- |
|  |  | **No** | **Yes** |  |
| **Number** | 6732 | 5384 | 1348 |  |
| **Age (years)** | 45.03 (0.32) | 46.21 (0.38) | 40.61 (0.40) | < 0.001 |
| **Sex (%)** |  |  |  | < 0.001 |
| Male | 3469 (51.59) | 2989 (55.28) | 480 (37.76) |  |
| Female | 3263 (48.41) | 2395 (44.72) | 868 (62.24) |  |
| **Race (%)** |  |  |  | 0.047 |
| Non-Hispanic White | 3986 (78.51) | 3259 (79.44) | 727 (75.06) |  |
| Non-Hispanic Black | 989 ( 7.47) | 770 (7.20) | 219 (8.48) |  |
| Mexican American | 1292 ( 5.67) | 994 (5.38) | 298 (6.75) |  |
| Other Race | 465 ( 8.35) | 361 (7.98) | 104 (9.71) |  |
| **Educational attainment (%)** |  |  |  | 0.002 |
| High school or less | 3190 (39.78) | 2498 (38.41) | 692 (45.09) |  |
| More than high school | 3536 (60.13) | 2881 (61.59) | 655 (54.91) |  |
| **Marital status (%)** |  |  |  | 0.136 |
| Married or living with partner | 4508 (68.88) | 3636 (69.44) | 872 (66.76) |  |
| Living alone | 2224 (31.12) | 1748 (30.56) | 476 (33.24) |  |
| **PIR (%)** |  |  |  | < 0.001 |
| Low | 984 (10.41) | 705 ( 8.89) | 279 (16.11) |  |
| Middle | 3511 (48.33) | 2777 (47.04) | 734 (53.16) |  |
| High | 2237 (41.26) | 1902 (44.07) | 335 (30.73) |  |
| **Drinking status (%)** |  |  |  | 0.026 |
| Never | 813 (10.53) | 640 (10.46) | 173 (10.81) |  |
| Mild | 2351 (36.18) | 1947 (37.44) | 404 (31.44) |  |
| Moderate | 1038 (17.40) | 812 (17.22) | 226 (18.07) |  |
| Heavy | 1280 (20.53) | 986 (20.03) | 294 (22.42) |  |
| Former | 1250 (15.35) | 999 (14.84) | 251 (17.26) |  |
| **CVD (%)** |  |  |  | 0.457 |
| Yes | 598 ( 6.80) | 501 (6.95) | 97 (6.23) |  |
| No | 6134 (93.20) | 4883 (93.05) | 1251 (93.77) |  |
| **Life's Simple 7 score components** |  |  |  |  |
| Physical activity score | 1.40 (0.01) | 1.40 (0.01) | 1.40 (0.02) | 0.868 |
| Smoking score | 1.26 (0.02) | 1.28 (0.02) | 1.18 (0.04) | 0.005 |
| Blood pressure score | 1.24 (0.01) | 1.21 (0.02) | 1.34 (0.02) | < 0.001 |
| Body mass index score | 1.04 (0.02) | 1.06 (0.02) | 0.98 (0.03) | 0.006 |
| Glucose score | 1.78 (0.01) | 1.78 (0.01) | 1.81 (0.01) | 0.007 |
| Cholesterol score | 1.27 (0.01) | 1.26 (0.01) | 1.31 (0.02) | 0.026 |
| Dietary intake score | 0.41 (0.01) | 0.44 (0.01) | 0.32 (0.01) | < 0.001 |
| **Life's Simple 7 score** | 8.41 (0.05) | 8.43 (0.06) | 8.36 (0.06) | 0.231 |

**Abbreviations:** PIR, family poverty income ratio; CVD, cardiovascular disease. aValues are weighted means (standardized errors) or number of participants (weighted percentages) unless otherwise indicated
